# Inhibition of BUB1 suppresses tumorigenesis of osteosarcoma via blocking of PI3K/Akt and ERK pathways

**DOI:** 10.1111/jcmm.16805

**Published:** 2021-08-01

**Authors:** Zhen Huang, Shenglin Wang, Hongxiang Wei, Hui Chen, Rongkai Shen, Renqin Lin, Xinwen Wang, Wenbin Lan, Rongjin Lin, Jianhua Lin

**Affiliations:** ^1^ Department of Rehabilitation The First Affiliated Hospital of Fujian Medical University Fuzhou China; ^2^ Department of Orthopedics The First Affiliated Hospital of Fujian Medical University Fuzhou China; ^3^ Fujian Orthopedics Research Institution The First Affiliated Hospital of Fujian Medical University Fuzhou China; ^4^ Department of Nephrology Shanghai East Hospital School of Medicine, Tongji University Shanghai China; ^5^ Department of Orthopedics, The people’s Hospital of Jiangmen City Southern Medical University Jiangmen China; ^6^ Department of Nursing The First Affiliated Hospital of Fujian Medical University Fuzhou China

**Keywords:** Akt, BAY 1816032, BUB1, ERK, lentivirus, osteosarcoma

## Abstract

Osteosarcoma (OS) is a primary malignant bone tumour that mainly affects teenagers, with patients displaying poor prognosis. Budding uninhibited by benzimidazoles 1 (BUB1), a type of serine/threonine kinase that is linked to pro‐tumorigenic phenomena, has not been well studied in OS. Hence, this study aimed to explore the role of BUB1 in OS. The expression of BUB1 in OS specimens and cell lines was assessed using immunohistochemistry and Western blot analysis. Univariate and multivariate analyses were applied to evaluate the impact of BUB1 on patient survival. Cell counting kit‐8, wound‐healing and Transwell assays, as well as flow cytometry, were used to investigate the influence of BUB1 inhibition on OS *in vitro*. Moreover, a tumour xenograft model was established to investigate the *in vivo* effect of BUB1 inhibition on OS tumour growth. Results showed that BUB1 was overexpressed in OS specimens and cell lines. Furthermore, BUB1 overexpression was closely associated with the poor clinical outcomes of patients with OS. Inhibition of BUB1 markedly suppressed cell proliferation and tumour growth, cell migration, invasion and induced cell apoptosis of OS by blocking the PI3K/Akt and ERK signalling pathways. Thus, our study suggested that overexpression of BUB1 protein contributed to poor survival of OS patients and that inhibition of BUB1 resulted in considerable anti‐tumour activity associated with proliferation, migration, invasion and apoptosis of OS.

## 1 INTRODUCTION

Osteosarcoma (OS), the most common primary invasive bone tumour, often occurs to young adults.^1^ The five‐year overall survival rate of patients with OS has improved from 20% to 70% because of the development of active therapies. However, approximately 30% of patients are prone to relapse or metastasis, and their prognosis remains poor because of the limited efficacy of the present treatment strategies.^2^ Thus, evaluation of efficient prognostic and therapeutic targets is essential to improve the poor clinical outcome of patients with OS.

Budding uninhibited by benzimidazoles 1 (BUB1) is a part of BUB family and mitotic arrest‐deficient (MAD) families of proteins.^3^ As a member of mitotic checkpoint serine/threonine kinases, BUB1 plays an important role in chromosome segregation.^4^ BUB1 contains three primary regions: a conserved N‐terminal region containing a kinetochore localization domain; an intermediate, non‐conserved region that acts as a scaffold for the recruitment of proteins; and a C‐terminal region that contains a catalytic serine/threonine kinase domain.^5^ BUB1 has been identified as an oncogene in diverse types of tumours.^6^ Overexpression of BUB1 is associated with tumour proliferation activity in human gastric carcinoma,^7^ papillary renal cell carcinoma^8^ and pancreatic ductal adenocarcinoma.^9^ Bioinformatic analyses by Sun et al.^10^ and Peng et al^11^ have shown that BUB1 likely plays a crucial role in OS; however, the results have not been confirmed in *in vitro* and *in vivo* experiments.

Therefore, this study aimed to further explore the role of BUB1 in OS by investigating the effects of BUB1 inhibition using either an inhibitor of BUB1, BAY 1816032 or lentivirus‐induced knockdown on the proliferation, migration, invasion, apoptosis and tumour growth of OS.

## 2 MATERIALS AND METHODS

### 2.1 Tissue samples

Forty‐one primary OS specimens and twenty limb‐fractured bone samples from patients who underwent surgical resection from 1 January 2011 to 31 December 2014 at the Department of Orthopedics of our hospital were selected retrospectively. All the participants included in the present study provided informed consent. Our research protocol complied with the Declaration of Helsinki ethical guidelines and was approved by the Institutional Review Board of the First Affiliated Hospital of Fujian Medical University.

### 2.2 Cell lines and culture

Human foetal osteoblastic cell line hFOB 1.19 and human OS cell lines MNNG/HOS and Saos2 were obtained from the Typical Culture Preservation Committee of the Chinese Academy of Sciences, Shanghai, China. MG63, 143B and U2OS cell lines were purchased from American Type Culture Collection (Manassas, VA, USA). hFOB 1.19 cells were maintained in DMEM/F12 medium containing 10% foetal bovine serum (FBS, Biological Industries, Israel) and 300 mg/ml neomycin (G418) in 5% CO_2_ atmosphere at 34°C. Saos2 cells were incubated in RPMI 1640 medium (Biological Industries, Israel) and U2OS, MNNG/HOS, 143B and MG63 cells in DMEM medium (Biological Industries, Israel) containing 10% FBS and 1% penicillin/streptomycin (Biological Industries, Israel) in an atmosphere of 5% CO_2_ at 37°C.

### 2.3 Protein extraction and Western blot analysis

Proteins were extracted using the Membrane and Cytosol Protein Extraction Kit (Beyotime, Shanghai, China), and their concentrations were quantified using the Bicinchoninic Acid Protein Assay Kit (Beyotime, Shanghai, China). Next, the proteins were subjected to 8% SDS‐polyacrylamide gel, blotted onto PVDF membranes (Millipore, Bedford, MA, USA) and then incubated in QuickBlock™ Blocking Buffer (Beyotime, Shanghai, China) for 15 min. Next, the membranes were incubated overnight at 4°C with primary antibodies against tubulin, BUB1, caspase 3, Bcl‐2, Akt, ERK, P‐Akt and P‐ERK, and all were purchased from Affinity Biosciences, Cincinnati, USA. The membranes were then incubated with secondary antibodies (Affinity) at room temperature for 1 h. The blots were finally developed using the Affinity ECL Kit (Affinity, Biosciences, Cincinnati, USA) and the FluorChem R detection system (ProteinSimple, USA).

### 2.4 Immunohistochemistry

The 61 samples collected from participants in this study were stained immunohistochemically with primary anti‐BUB1 antibody (Affinity, Biosciences, Cincinnati, USA) using the PV9000 immunohistochemical kit (Origene Technologies, Inc., Beijing, China). Prior to that, the site of tumour in the specimens was identified using haematoxylin‐eosin (HE) staining. Two independent pathologists evaluated the staining results. The labelling index of BUB1 expression was scored as 0 to 3 based on staining intensities: negative, 0; weakly positive, 1; moderately positive, 2; and strongly positive, 3. The mean percentage based on staining from 10 random high‐power fields of positive tumour cells was also scored as 1 to 3 as follows: 1, <25%; 2, 25%–75%; and 3, >75%. Total scores were determined based on the intensity and percentage of positive staining in cancer cells. Overexpression was defined as a score >2, and low expression was defined as a score ≤2.^12^

### 2.5 BUB1 knockdown cell line

Cell lines were infected with a knockdown lentivirus (sh‐BUB1, TCCTACACTTCCTGATATT) and the corresponding negative control lentivirus (sh‐NC, TTCTCCGAACGTGTCACGT), purchased from GeneChem (Shanghai, China). The cells were plated into 6‐well plates at 1 × 10^5^ cells/well and incubated overnight. Next, the medium was replaced with 1 ml fresh medium containing an appropriate amount of virus suspension. After 24 h, the virus‐containing medium was replaced with fresh medium and the plates were incubated for another 48 h. Stably transfected clones were screened using puromycin. Transfection efficiency was confirmed by Western blot analysis.

### 2.6 Cell viability assay

Cells were maintained at a density of 2 × 10^3^ cells/well in 96‐well plates overnight and then incubated with various concentrations of BAY 1816032 or DMSO for 72 h. Cells infected with lentivirus were incubated in 96‐well plates at the same density mention above for 24, 48, 72 or 96 h. Subsequently, after adding 100 μl medium containing 10 μl of CCK‐8 solution (New Cell & Molecular Biotech, Co., Ltd, China) per well, the cells were then incubated at 37℃ for 2 h. Absorbance values were measured at 450nm using a microplate reader (ELx800; Bio‐Tek, USA).

### 2.7 Wound‐healing assay

OS cells or lentivirus‐infected cells were plated at a density of 5 × 10^5^ cells/well in 6‐well plates and incubated for 24 h. The cell monolayers were then scraped using a 200‐μl sterile pipette tip. To investigate the effect of BAY 1816032 on OS, the cells were treated with 2.5, 5, 10 µM BAY 1816032 or DMSO in a medium without FBS. sh‐BUB1 and sh‐NC cells were also cultured in the same medium. Images were obtained at 0, 24 and 48 h using an inverted optical microscope equipped with Zen Imaging software (Carl Zeiss, Oberkochen, Germany).

### 2.8 Transwell assay

Transwell assay was performed in an 8‐μm pore size chamber (Corning Inc., Corning, NY, USA), in the absence (migration) or presence (invasion) of Matrigel (BD Biosciences, Franklin Lakes, NJ, USA) in the upper chamber. A total of 5 × 10^4^ cells/well pre‐treated with either BAY 1816032 or lentivirus were seeded in the upper chamber in a culture medium that did not contain serum, and 600 μl of complete medium was added to the lower chamber. After incubation for 24 h, non‐penetrated cells were removed, and the migrated or invasive cells in the lower chamber were fixed with methanol and stained with 0.1% crystal violet (Beyotime, Shanghai, China). The numbers of migrated or invasive cells were counted under an inverted optical microscope.

### 2.9 Flow cytometry

Saos2 and U2OS cells were cultured in 6‐well plates at 2.0 × 10^5^ overnight and treated with different concentrations of BAY 1816032 or DMSO for 72 h. sh‐BUB1 and sh‐NC cells were cultured in 6‐well plates at the density mentioned above for 72 h. Cell apoptosis was detected employing the Annexin V‐FITC Apoptosis Detection Kit (Beyotime, Shanghai, China) according to the manufacturer's instructions. Results were analysed using a flow cytometer (BD Biosciences, Franklin Lakes, NJ, USA) and measured using the FlowJo software (BD Biosciences).

### 2.10 Animal experiments

Animal experiments were approved by the Committee of Animal Ethics of our hospital and conducted in accordance with the Guide for the Care and Use of Animals for research purposes. Nude mice (BALB/c, 4–6 weeks old) were purchased from SLAC, Shanghai, China. The lentivirus‐knockdown effect of BUB1 on tumour growth was investigated by injecting Saos2 cells with sh‐BUB1 or sh‐NC cells (2 × 10^6^ per mouse) into the left subcutaneous region of nude mice. Tumour sizes were measured weekly after transplantation for seven weeks. Similarly, the effect of BUB1 inhibitor was investigated by injecting Saos2 cells (2 × 10^6^ per mouse) into the left subcutaneous region of nude mice. A week later, mice with similar tumour sizes were divided into the control group and the BAY 1816032 groups and were injected DMSO and BAY 1816032, respectively, via the tail vein. The treatment was carried out every day for the first three days and every second day for the subsequent 20 days, with tumour sizes being measured weekly for seven weeks.

### 2.11 Statistical analysis

SPSS 19.0 software (SPSS Inc., Chicago, USA) was used for statistical analysis. The chi‐square test or Fisher's exact test was performed to investigate the difference in BUB1 protein expression between OS and normal bone tissues, as well as the relationship between BUB1 protein expression and clinicopathological parameters. Differences in survival status were measured by log‐rank test and Kaplan‐Meier survival plots. Cox proportional hazards model was performed on the parameters that displayed significantly different in the univariate analysis. Difference between groups was analysed using Student's *t* test; data are presented as mean ± SD. A *p* < 0.05 was considered to be statistically significant.

## 3 RESULTS

### 3.1 BUB1 expression is up‐regulated in OS tissues and cell lines

**FIGURE 1:**
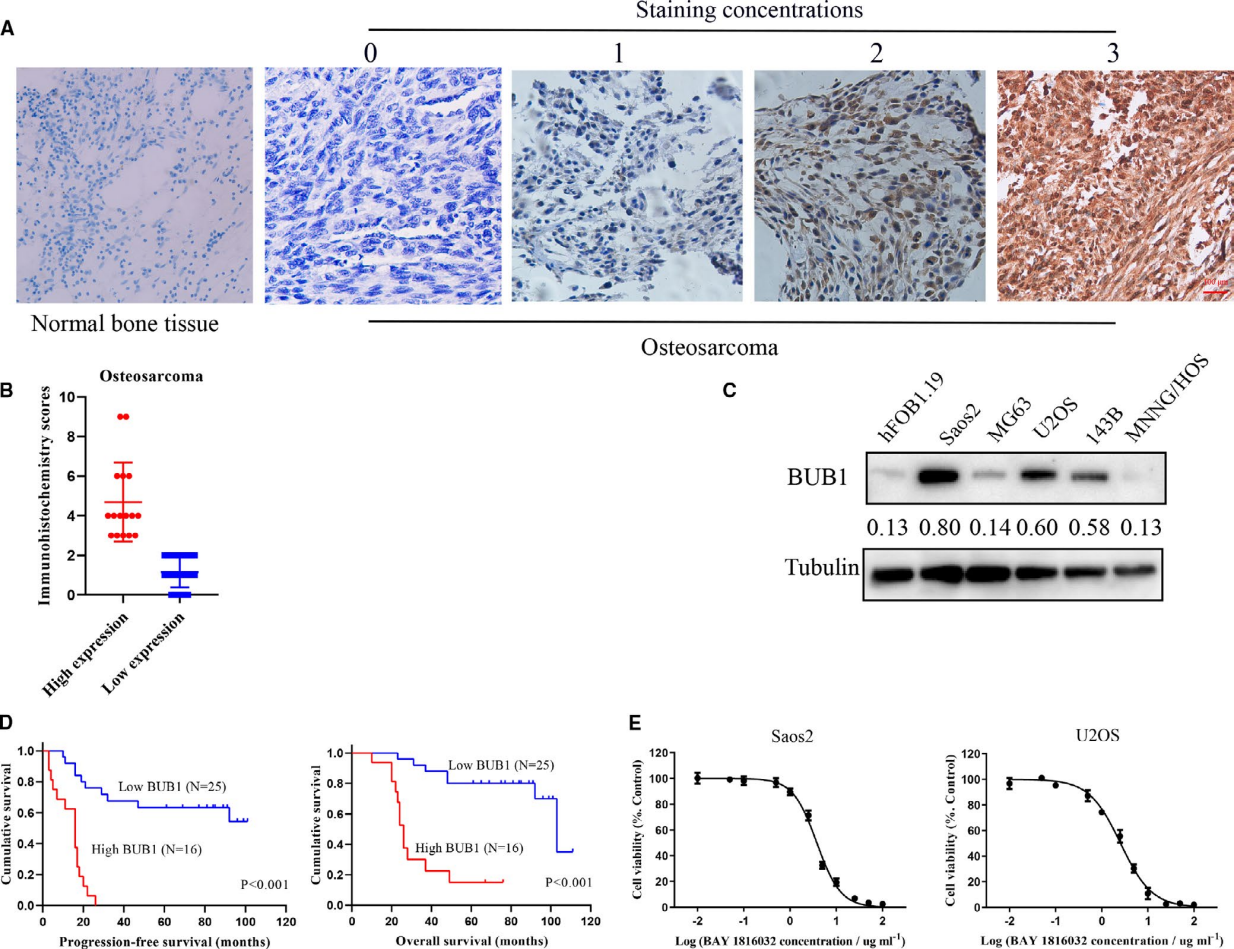
Expression of BUB1 protein in osteosarcoma (OS) tissues and cell lines. A, Representative images of different staining intensities of BUB1 protein in OS and normal bone tissues. B, Immunohistochemistry scores of OS tissues. C, Representative images showing the up‐regulated expression of BUB1 protein in Saos2, U2OS and 143B cells; the ratio calculation was detected by using grey analysis. D, Significant differences in progression‐free survival (PFS) (*p* < 0.001) and overall survival (*p* < 0.001) time between OS patients with high and low expression of BUB1. E, Cell viability was markedly reduced by incubating cells with BAY 1816032 for 72 h in Saos2 and U2OS cells.

Immunohistochemistry results demonstrated that the BUB1 protein was overexpressed in 39.02% (16/41) of OS tissues but not in the normal bone tissues (Figure 1A,B). We further examined the expression of BUB1 in 143B, U2OS, MNNG/HOS, MG63, Saos2 and hFOB 1.19 cells using Western blot analysis. BUB1 expression was up‐regulated in Saos2, U2OS and 143B cells, with the highest levels being expressed in U2OS and Saos2 cells (Figure 1C).

### 3.2 BUB1 expression is associated with clinicopathological characteristics and clinical outcome

Among the clinicopathological parameters, including sex, age, tumour size, tumour location, histologic subtype, Enneking staging, response to chemotherapy and distant metastasis. Response to chemotherapy (*p* < 0.001) and distant metastasis (*p* = 0.036) were found to be closely related to BUB1 protein overexpression (Table 1). Independent prognostic value of BUB1 protein in OS was evaluated by performing univariate and multivariate analyses to investigate the relationships of BUB1 protein overexpression with progression‐free survival (PFS) time and overall survival (OS) time in patients with OS. As shown in Figure 1D and Table 2, univariate analysis revealed that overexpression of BUB1 was related to poor PFS (*p* < 0.001) and OS (*p* < 0.001) in patients with OS. Moreover, response to chemotherapy (PFS, *p* < 0.001; OS, *p* < 0.001) and distant metastasis (PFS, *p* < 0.001; OS, *p* = 0.002) showed a significant influence on the poor prognosis. Multivariate analysis (Table 3) indicated that BUB1 protein overexpression was closely related to poor PFS [hazard ratio (HR) = 3.442, *p* = 0.031] and OS (HR = 4.611, *p* = 0.014) time in OS. Therefore, BUB1 protein overexpression can be considered as an independently prognostic biomarker for OS.

**TABLE 1:** Association between BUB1 expression and clinicopathological characteristics in osteosarcoma

Clinicopathological data	Case number	High expression (*n* = 16)	Low expression (*n* = 25)	*p*‐value
Sex				
Male	27	8	19	0.087
Female	14	8	6	
Age (years)				
<18	14	7	7	0.300
≥18	27	9	18	
Tumour size (cm)				
<8	24	11	13	0.288
≥8	17	5	12	
Tumour location				
Tibia and Femur	23	8	15	0.529
Others	18	8	10	
Histologic subtype				
Conventional	34	13	21	0.819
Others	7	3	4	
Enneking staging				
I‐IIA	10	4	6	0.942
IIB	31	12	19	
Response to chemotherapy				
Poor	17	13	4	<0.001
Good	24	3	21	
Distant metastasis				
Yes	15	9	6	0.036
No	26	7	19	

**TABLE 2:** Univariate analysis of BUB1 expression and osteosarcoma patient survival

Characteristics	Case number	Progression‐free survival (months)				Overall survival (months)			
		Mean	SD	95% CI	*p*‐value	Mean	SD	95% CI	*p*‐value
Sex									
Male	27	54.015	7.943	38.447–69.583	0.134	75.107	6.681	62.012–88.202	0.293
Female	14	33.857	9.586	15.068–52.646		60.516	10.726	39.493–81.539	
Age (years)									
<18	14	34.357	8.839	17.033–51.682	0.225	57.643	9.062	39.880–75.405	0.216
≥18	27	53.333	8.101	37.454–69.212		76.005	7.012	62.262–89.749	
Tumour size (cm)									
<8	24	47.979	8.262	31.785–64.173	0.821	69.685	7.759	54.477–84.893	0.880
≥8	17	46.412	10.024	26.765–66059		70.886	8.737	53.762–88.011	
Tumour location									
Tibia or femur	23	57.130	8.820	39.843–74.418	0.053	78.026	7.194	63.926–92.127	0.080
Others	18	34.593	8.174	18.571–50.615		59.944	8.804	42.688–77.201	
Histologic subtype									
Conventional	34	48.603	7.017	34.850–62.356	0.717	68.804	6.365	56.329–81.280	0.839
Others	7	40.286	14.703	11.467–69.105		71.667	13.220	45.756–97.577	
Enneking staging									
I‐IIA	10	57.375	13.322	31.264–83.486	0.481	76.614	10.986	55.083–98.146	0.706
IIB	31	41.161	7.177	30.094–58.229		68.846	6.772	55.572–82.120	
Response to chemotherapy									
Poor	17	16.471	2.518	11.536–21.405	< 0.001	38.752	6.300	26.404–51.100	< 0.001
Good	24	68.946	8.208	52.859–85.034		86.052	5.680	74.919–97.185	
Distant metastasis									
Yes	15	16.267	2.783	10.811–21.722	< 0.001	42.071	5.469	31.352–52.791	0.002
No	26	65.027	8.039	49.271–80.784		83.228	6.689	70.117–96.338	
BUB1 expression									
High	16	13.563	1.793	10.047–17.078	< 0.001	33.707	5.297	23.324–44.089	< 0.001
Low	25	68.709	7.745	53.528–83.889		88.780	5.312	78.369–99.191	

**TABLE 3:** Multivariate analysis of BUB1 expression and osteosarcoma patient survival

Characteristics	Comparison	Progression‐free survival (months)			Overall survival (months)		
		HR	95% CI	*p*‐value	HR	95% CI	*p*‐value
BUB	High vs. low	3.442	1.122–10.557	0.031	4.611	1.362–15.618	0.014
Response to chemotherapy	Poor vs. good	2.209	0.758–6.434	0.146	2.305	0.739–7.185	0.150
Distant metastasis	Yes vs. no	2.160	0.868–5.377	0.098	1.965	0.682–5.657	0.211

### 3.3 BUB1 inhibition suppresses cell viability, migration and invasion of OS cells *in vitro*

**FIGURE 2:**
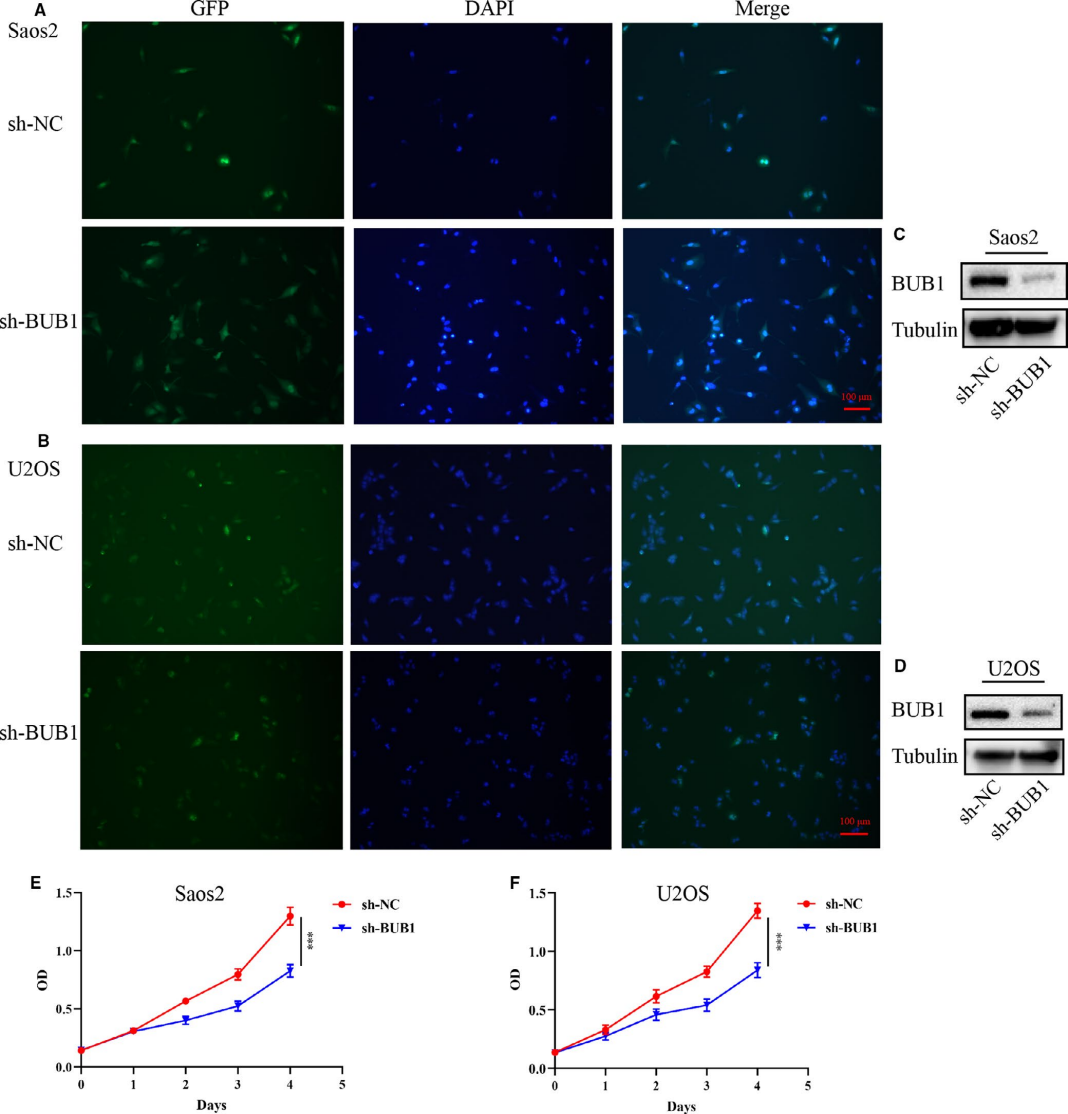
Effect of BUB1 knockdown on the proliferation of OS cells. A, B, Interference efficiency of stably transfected Saos2 or U2OS cells. C, D, Western blotting to verify the expression of BUB1 gene in lentivirus‐transfected Saos2 or U2OS cells. E, F, Effect of BUB1 gene interference on the proliferation of Saos2 or U2OS cells detected by cell counting kit‐8 (CCK‐8). ***p < 0.001.

**FIGURE 3:**
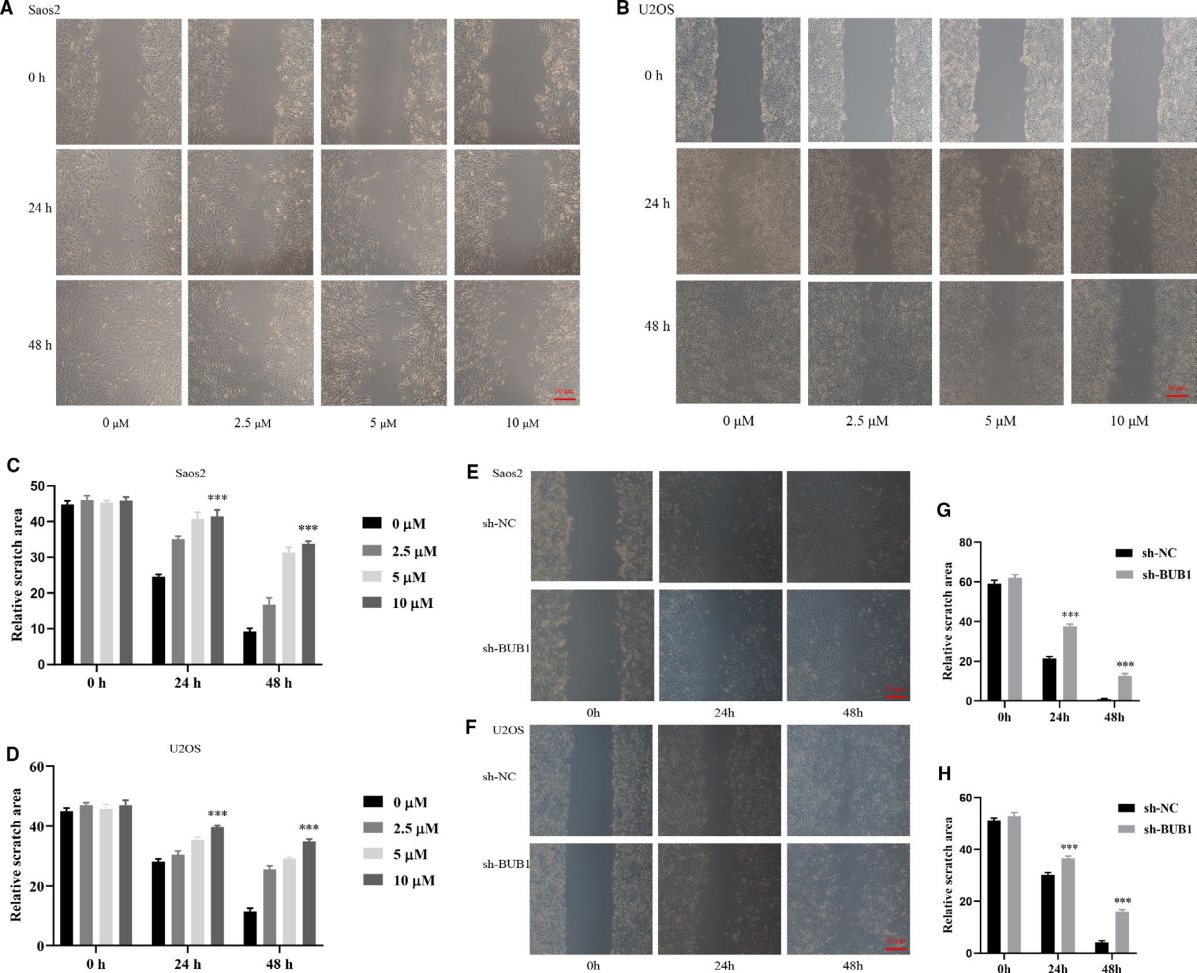
Effect of BUB1 inhibition on the relative scratch areas of Saos2 and U2OS cells using a wound‐healing assay. A‐D, Markedly suppressed relative scratch areas of Saos2 and U2OS cells by treatment with BAY 1816032 for 24 and 48 h. E‐H, Significantly reduced scratch areas of Saos2 and U2OS cells after BUB1 gene knockdown. ***p < 0.001.

**FIGURE 4:**
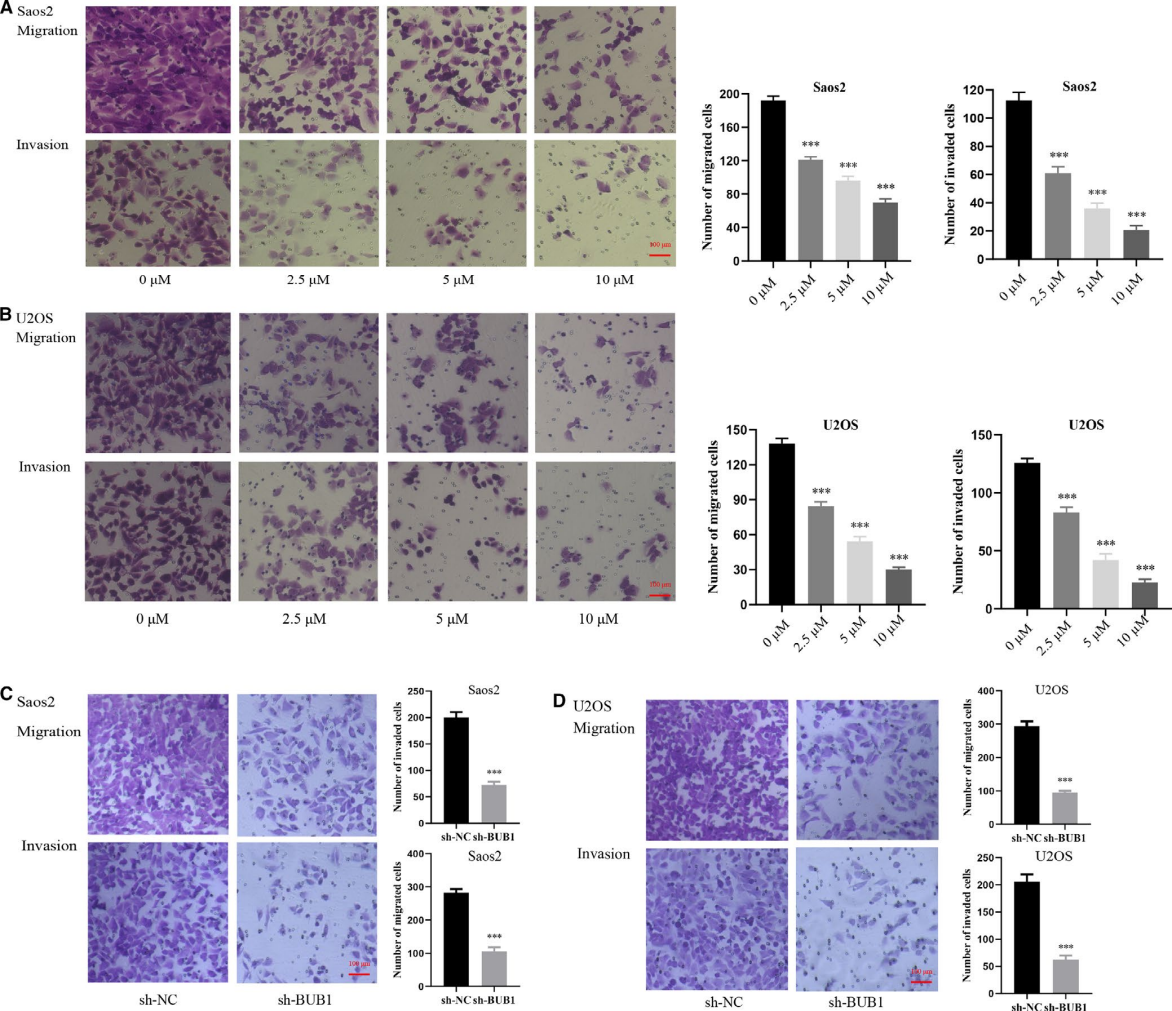
Effect of BUB1 inhibition on the migration and invasion of Saos2 and U2OS cells using a Transwell assay. A, B, Significantly decreased numbers of migrated and invaded Saos2 and U2OS cells with increasing concentrations of BAY 1816032. C, D, Significantly decreased numbers of migrated and invaded Saos2 and U2OS cells after BUB1 gene knockdown. ***p < 0.001.

Treatment of OS cells with increased concentrations of BAY 1816032 decreased the viability of tumour cells, with IC50 values of 3.80 μM in Saos2 cells and 2.54 μM in U2OS cell, as shown in Figure 1E. Simultaneously, Saos2 and U2OS cells with relatively high BUB1 expression were transfected with BUB1 knockdown lentivirus (sh‐BUB1) or corresponding negative control lentivirus (sh‐NC) (Figure 2A,B). The sh‐BUB1 cells, which showed remarkably decreased BUB1 expression, were found to have decreased cell viability (Figure 2C–F). To further investigate the effect of BUB1 inhibition on the migration and invasion of OS cells, we performed wound‐healing and Transwell assays. Results of the wound‐healing assay demonstrated that the relative scratch area significantly decreased, depending on the use of increasing concentration and treatment time of BAY 1816032 in Saos2 and U2OS cells (Figure 3A,B). Similarly, lentivirus intervention also suppressed the migration ability of OS cells (Figure 3C,D). Results of the Transwell assay indicated that both the migration and invasion abilities were reduced notably in Saos2 and U2OS cells because of treatment with BAY 1816032 (Figure 4A,B) or by lentivirus‐induced knockdown of BUB1 (Figure 4C,D).

### 3.4 BUB1 inhibition promotes cell apoptosis of OS cells *in vitro*

**FIGURE 5:**
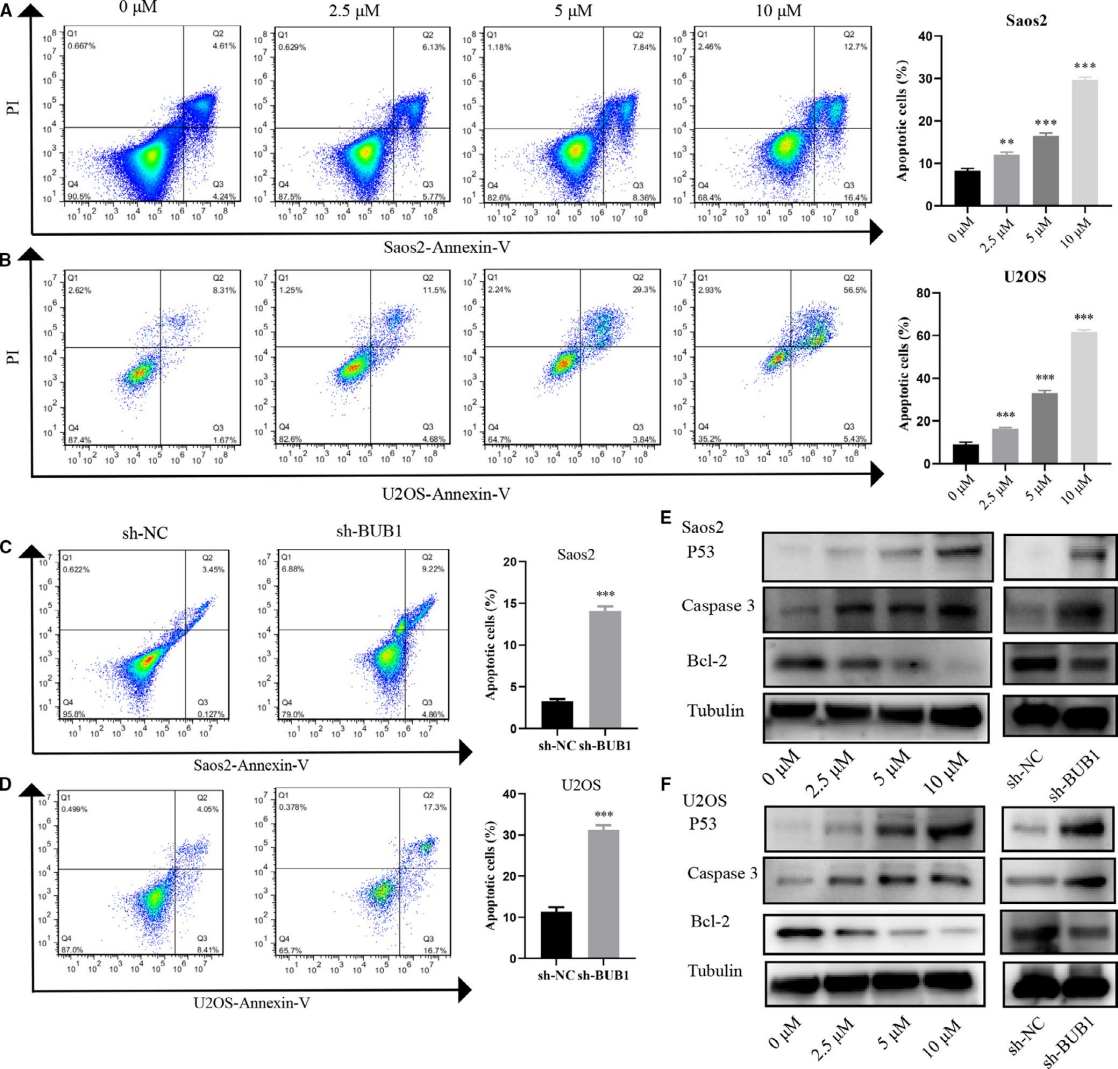
Effect of BUB1 inhibition on the cell apoptosis and apoptosis‐related proteins. A, B, Increased total apoptosis rate in Saos2 and U2OS cells after treatment with different concentrations of BAY 1816032. C, D, Increased total apoptosis rate in Saos2 and U2OS cells with BUB1 gene knockdown. E, F, Expression of P53, caspase 3 and Bcl‐2 proteins in Saos2 and U2OS cells following treatment with BAY 1816032 and BUB1 gene knockdown. **p < 0.01, ***p < 0.001.

To explore cell death caused by inhibition of BUB1, we used the Annexin V‐FITC Kit to assess the apoptosis rate after the treatment for 72 h using BAY 1816032 or lentivirus transfection. Inhibition of BUB1 by BAY 1816032 or lentivirus‐induced knockdown of BUB1 induces late apoptosis of OS cells. Treatment with 2.5 μM BAY 1816032 resulted in an increase in apoptosis rate by approximately 3% in Saos2 and by 7% in U2OS cells. Treatment with 5 μM BAY 1816032 resulted in an apoptosis rate of approximately 7% in Saos2 and 20% in U2OS cells. Furthermore, 10 μM BAY 1816032 increased the total apoptosis rate by 20% in Saos2 and by 50% in U2OS cells (Figure 5A,B). Similarly, lentivirus‐induced BUB1 knockdown promoted cell apoptosis by approximately 10% in Saos2 and 20% in U2OS cells (Figure 5C,D). Moreover, Western blot analysis of apoptosis‐related proteins demonstrated markedly increased P53 and caspase 3 expression and decreased Bcl‐2 expression following treatment with BAY 1816032 or intervention using lentivirus. Thus, these results suggest that BUB1 inhibition had a significant facilitated effect on the apoptosis of OS cells.

### 3.5 BUB1 inhibition suppresses tumour growth of OS *in vivo*

**FIGURE 6:**
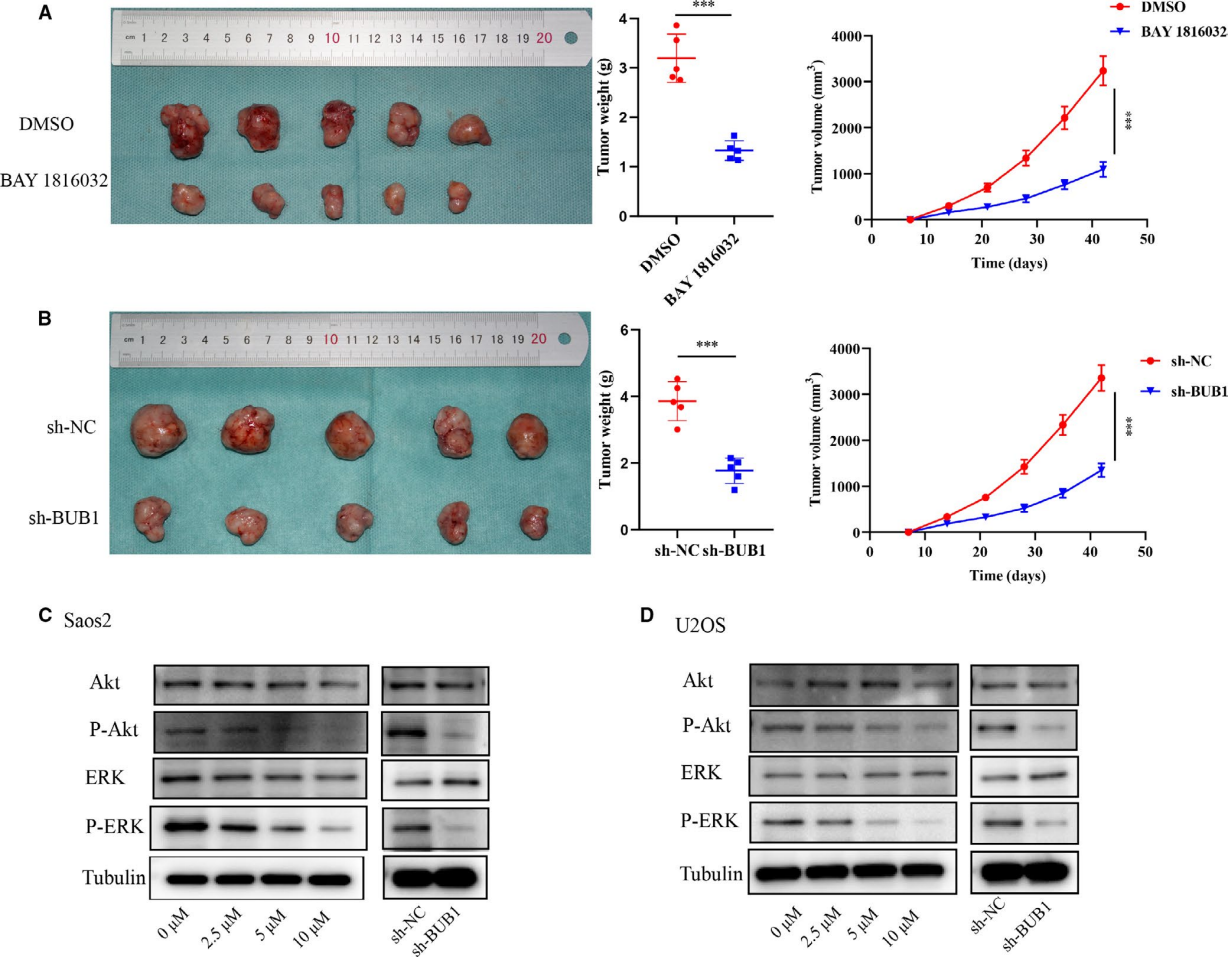
The effect of BUB1 inhibition on tumour growth of OS *in vivo* and the related mechanism. A, Tumour images and the differences in tumour weights and tumour volumes in the DMSO and BAY 1816032‐treated groups. B, Tumour images and the differences in tumour weights and tumour volumes in sh‐NC and sh‐BUB1‐treated groups. C, D, Total and phosphorylated expressions of Akt and ERK proteins in Saos2 and U2OS cells by Western blot analysis after BAY 1816032 treatment and BUB1 knockdown. Tubulin was used as internal loading control. ***p < 0.001.

To evaluate the effect of BUB1 suppression and BUB1 gene knockdown on tumour growth of OS *in vivo*, an OS xenograft model was established in nude mice. We found that both BAY 1816032 treatment and BUB1 knockdown resulted in the regression tumour growth, as demonstrated by decreased tumour volumes and weights of mice in the experiment group (Figure 6A,B).

### 3.6 BUB1 affects the biological behaviours of OS via phosphatidylinositol 3 kinase (P13K)/Akt and ERK pathways

To investigate the effects of BUB1 on OS, we assessed the activity of PI3K/Akt and ERK pathways in Saos2 and U2OS cells by Western blot analysis following inhibition of BUB1. As shown in Figure 6C,D, the expression of P‐Akt and P‐ERK proteins was reduced following treatment with BAY 1816032, similarly, BUB1 knockdown suppressed P‐Akt and P‐ERK proteins. However, the expressions of Akt and ERK were not significantly changed by BUB1 inhibition in Saos2 and U2OS cells. Therefore, PI3K/Akt and ERK pathways may be involved in the effect of BUB1 on OS cells.

## 4 DISCUSSION

OS, which originates from mesenchymal tissue, is one of the most aggressive primary bone tumours among children and adolescents.^13^ Moreover, the survival status of patients with metastatic or relapsed disease has not improved in the past two decades.^14^ Therefore, new biomarkers and therapeutic targets for OS should be urgently identified to improve clinical outcomes of patients. Our study showed that the BUB1 protein was overexpressed in OS, which was closely related to adverse clinicopathological parameters and poor prognosis of OS. Treatment with BAY 1816032, an inhibitor of BUB1, or knockdown of BUB1 could suppress the biological activity of OS related to cell proliferation, apoptotic tolerance, migration and invasion *in vitro* and tumour growth *in vivo*, with the involvement of PI3K/Akt and ERK pathways. These results suggested that BUB1 could serve as an independent biomarker for the prognosis of OS and also as an effective therapeutic target for OS treatment.

Previous research has confirmed that BUB1 could be viewed as a clinical biomarker for adverse prognosis and a potential therapeutic target in various tumours,^15^, ^16^ including aggressive colorectal cancer,^17^ breast cancer^18^ and glioblastoma.^19^ Consistent with these studies, we showed that the overexpression of the BUB1 protein was associated with a poor response to chemotherapy and distant metastasis of OS. Using Cox regression analysis, we further demonstrated that the overexpression of BUB1 could serve as an independent biomarker for predicting the poor PFS and overall survival time of patient with OS.

Abnormal expression of BUB1 influences the function of spindle checkpoints, thereby leading to chromosomal instability during mitosis.^20^ A complete loss of spindle checkpoints is frequently lethal, and reducing the potential cooperation of such checkpoints with other genes could facilitate tumorigenesis.^21^ A broad and unified analysis based on GEO data set showed that BUB1 is associated with tumorigenesis and is a potential diagnostic and therapeutic target for OS.^10^ Peng et al^11^ performed bioinformatic analysis to demonstrate that BUB1 was a potential gene associated with conventional OS and might have a crucial role on the pathogenesis of this disease. We performed *in vitro* and *in vivo* experiments, further confirming that inhibition of BUB1 in OS could suppress cell proliferation, migration and invasion, and tumour growth, as well as promote cell apoptosis. Interestingly, we found that the expression of P53 protein and the numbers of apoptotic cells were higher in U2OS cell line than that in Saos2 cell line following treatment with BAY 1816032 and BUB1 gene knockdown. The expression of P53 protein can induce cell apoptosis in osteosarcoma.^22^, ^23^ A previous study confirmed that the P53 protein was not present in Saos2 cells^24^ but was found in U2OS cells.^25^ Our results suggest that apoptotic cells were present in greater numbers in U2OS cells than in Saos2 cells, when treated with BAY 1816032 or lentivirus‐induced knockdown of BUB1.

BAY 1816032 is a novel, bioavailable inhibitor of catalytic activity of the mitotic checkpoint protein BUB1, which plays a role in centromere cohesion and attachment error correction.^26^ Depletion of BUB1 in lung adenocarcinoma and breast cancer results in the abrogation of downstream effectors of phosphorylated PI3K/Akt and ERK, thereby leading to the suppression of cell proliferation, migration and invasion.^27^, ^28^ PI3K/Akt and ERK pathways are crucial oncogenic pathways in the progression of OS. Zhang et al^29^ reported that CircRNA hsa_circ_0005909 promotes the development of OS by enhancing the activation of the PI3K/Akt and ERK pathways. Wang et al^30^ demonstrated that hyperactivation of epidermal growth factor receptor (EGFR) promotes cell proliferation and metastasis by activating PI3K/Akt and ERK pathways in OS. Therefore, we explored the involvement of PI3K/Akt and ERK signalling pathways in the effects of BUB1 inhibition on OS. The results verified that because of treatment with BAY 1816032 or lentivirus‐induced knockdown of BUB1, phosphorylation levels of Akt and ERK proteins were reduced. Therefore, our results show that BUB1 may be an important therapeutic target in OS.

Nevertheless, certain limitations of our study should be taken into account. The case numbers and follow‐up time in our research were insufficient; a longer follow‐up time is required, and studies have to be performed on a larger scale. Our subsequent work will further focus on other potential roles and related mechanisms of BUB1 in OS.

## 5 CONCLUSIONS

This study reports up‐regulation of BUB1 in OS, which is closely related to the adverse clinical outcomes of patients with OS. Suppression of BUB1 protein could significantly reduce cell proliferation, invasion and migration, promote apoptosis of OS *in vitro* and inhibit tumour growth *in vivo*. Therefore, our study may provide a new therapeutic target for the treatment of OS, which may improve the current stagnant survival of patients. However, further investigations are required to confirm the therapeutic effect of BUB1 on OS.

## CONFLICT OF INTEREST

None authors have any conflict of interest.

## AUTHOR CONTRIBUTIONS

**Zhen Huang:** Conceptualization (equal); Data curation (equal); Writing‐original draft (equal); Writing‐review & editing (equal). **Shenglin Wang:** Data curation (equal); Writing‐review & editing (equal). **Hongxiang Wei:** Formal analysis (equal); Methodology (equal). **Hui Chen:** Formal analysis (equal); Methodology (equal). **Rongkai Shen:** Formal analysis (equal); Methodology (equal). **Renqin Lin:** Formal analysis (equal); Methodology (equal). **Xinwen Wang:** Formal analysis (equal); Methodology (equal). **Wenbin Lan:** Formal analysis (equal); Methodology (equal). **Rongjiin Lin:** Conceptualization (equal); Data curation (equal); Funding acquisition (equal); Methodology (equal); Validation (equal); Writing‐original draft (equal); Writing‐review & editing (equal). **Jianhua Lin:** Conceptualization (equal); Data curation (equal); Funding acquisition (equal); Methodology (equal); Validation (equal); Writing‐original draft (equal); Writing‐review & editing (equal).
